# The Schnauzenorgan-response of *Gnathonemus petersii*

**DOI:** 10.1186/1742-9994-6-21

**Published:** 2009-09-22

**Authors:** Jacob Engelmann, Sabine Nöbel, Timo Röver, Gerhard von der Emde

**Affiliations:** 1 University of Bonn, Institute for Zoology, Endenicher Allee 11-13, 53115 Bonn, Germany

## Abstract

**Background:**

Electric fish navigate and explore their dark and turbid environment with a specialised electric sense. This active electrolocation involves the generation and perception of an electric signal and fish have proven to be useful model systems for the investigation of sensory-motor interactions. A well studied example is the elephantnose fish, *Gnathonemus petersii*, which has a characteristic and unique elongated chin covered with hundreds of electroreceptor organs. This highly moveable so-called Schnauzenorgan constitutes the main fovea of the active electrosensory system. Here we present first evidence for a sensory-motor loop relating active electrical sensing to active motor exploration of the environment.

**Results:**

Both anatomical and behavioural evidence have shown that the moveable Schnauzenorgan is crucial for prey localization. Here we show for the first time that a motor response (Schnauzenorgan-response, SOR) can be elicited by novel electrosensory stimuli. The SOR could be triggered with highest reliability by novel electrical stimuli near the Schnauzenorgan and, to a lesser extend, near the head of the animal. The probability of evoking the response depended on the magnitude of the amplitude change of the electric input, with bigger changes eliciting SORs more reliably. Similarly, increasing the distance of the stimulus reduced the response. In this respect the SOR is comparable to the well described novelty response, a transient acceleration of the production rate of electric signals, although the latter occurs at a shorter delay and can also be evoked by non-electrical stimuli.

**Conclusion:**

Our experiments show a novel motor response that is mediated by the active electric sense of *Gnathonemus petersii*. This response will allow a detailed analysis of the neural system underlying direct interaction between sensory and motor processes in future experiments.

## Background

In the course of evolution many different sensory systems and sensory receptors have developed. One of the rather unique sensory systems is that of active electrolocation and electro-communication found in Mormyriform and Gymnotiform weakly electric fishes from Africa and South America, respectively. During active electrolocation mormyrids emit and simultaneously perceive electric signals, which enable them to detect and analyse nearby objects. This is considered as an adaptation enabling electric fish to extend their activity to the hours of darkness, since the dependence on vision is expected to be reduced.

The electric field of *G. petersii *is generated by a synchronous discharge of an electric organ. Each electric organ discharge (EOD) has a duration of roughly 400 μs, and at rest EODs are elicited 3 to 8 times per second [1,2]. This discharge frequency varies between 1 and 100 Hz and depends on the behavioural context. The electric field that surrounds the animal during each EOD is optimized for electrolocation around the head as the field has the highest coherence and impinges at an angle of almost 90° onto the receptors [3]. Since the amplitude of this field drastically declines with distance, active electrolocation is generally confined to the range of one body length of the animal [4].

Both ampullary and tuberous electroreceptor organs are devoted to the detection of electrical fields. Ampullary electroreceptors are extremely sensitive to low frequency fields of biotic or abiotic origin and are generally used in the context of passive electrolocation [5]. In contrast, tuberous electroreceptor organs are involved in active electrolocation (Mormyromasts, [6,7]) or electro-communication (Knollenorgans, [8]). Central filtering mechanisms enhance sensory information conveyed by the Mormyromasts in response to the self-generated EOD only, whereas Knollenorgan input is selectively filtered centrally such that secondary neurones are very sensitive to the EODs of conspecifics [9].

In this study we are mainly interested in possible links between (motor) behaviour and electrolocation. It has been shown that the Mormyromast system is important for foraging and orientation [10,11]. Fish can perceive a wealth of information form their 'electrical' world, including parameters such as size and distance of objects and the differentiation of various object properties, like capacitive and resistive electrical properties [for review see: [12]].

The main stimulus parameters utilised by the animals are phase and amplitude of the EOD. Briefly, the EOD can be modulated in amplitude as well as in its waveform by a nearby object. Local EOD amplitude is determined by the resistance of an object, with low resistance objects causing an increase in the local EOD amplitude, while non-conductors decrease the stimulus amplitude [13]. Capacitive objects of a certain range of capacitances change the EOD-amplitude and additionally distort the EOD waveform [14,15].

A well known behaviour linked to electro-perception in *G. petersii *is a sudden and transient increase in the EOD rate (shortening of inter-EOD intervals) when a nearby object is suddenly altered in its properties. This so-called „novelty response‟ [6] is found both in Mormyriform and Gymnotiform weakly electric fishes [16,17]. The novelty response can be regarded as an active electrical orientation mechanism in response to new sensory input [18]. This response can be evoked by electrical [19-23], acoustical [24], visual [17] and mechanosensory lateral-line stimuli [25] as well as multi-model stimuli [26].

Anatomical data indicate a special role of the Schnauzenorgan (SO) and the head during active electrolocation [3,27]. Based on these data and corroborating behavioural observations, the SO and the nasal region are believed to be functionally equivalent to two distinct electrical "foveae". One of the behaviours that support a foveal function of the SO are oscillating movements during foraging, i.e., the Schnauzenorgan is bent in rapid movements perpendicular to the animal's longitudinal axis [28]; Hollmann et al, in preparation). In addition, sudden movements of the Schnauzenorgan can be observed when novel objects are detected. A key question in the current paper is whether SO movements are linked to electro-perception and if so, which type of stimuli might evoke this behaviour.

Given the fast rhythmic movements of the SO during foraging, we here investigate the hypothesis that these movements, like the novelty response extend the reach of electrolocation, especially during the detection and analysis of novel stimuli. We confirm this hypothesis and further characterise this motor-response by determining which receptors contribute to it, how it is linked to the novelty response and by determining the sensitivity of the response for stimuli at various body regions.

## Results

### Schnauzenorgan response

In the following we describe the movements of the Schnauzenorgan in response to changes in the impedance of dipole objects. Following a general description of the responses to our standard stimulus paradigm, we present in detail how these responses depend on the distance and position of the dipole object, and how the SOR is linked to the novelty response.

**Figure 1:**
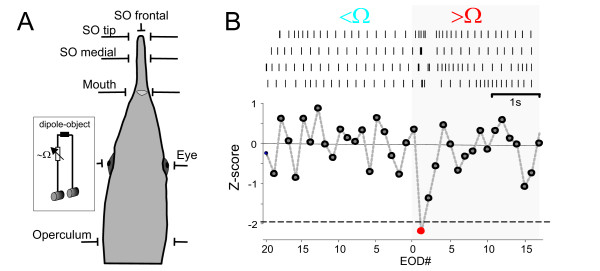
**Set-up**. **A**. Schematic illustrating the five paired lateral positions and the frontal position of the dipole-object (see inset). Distances from skin were varied between 1, 3, 5, 7, 10, 12 and 15 mm. **B**. Example for data extraction for the novelty response. The top shows EODs in response to four consecutive switches between a low resistance (shunt) and a high resistance as a raster plot. Following normalisation by the mean and variance (z-score), a significant acceleration of the EOD rate is apparent in the bottom plot (red dot). Responses were scored as significant if z-scores below -1.96 were achieved within 5 EODs after the switch (p < 0.05).

**Figure 2:**
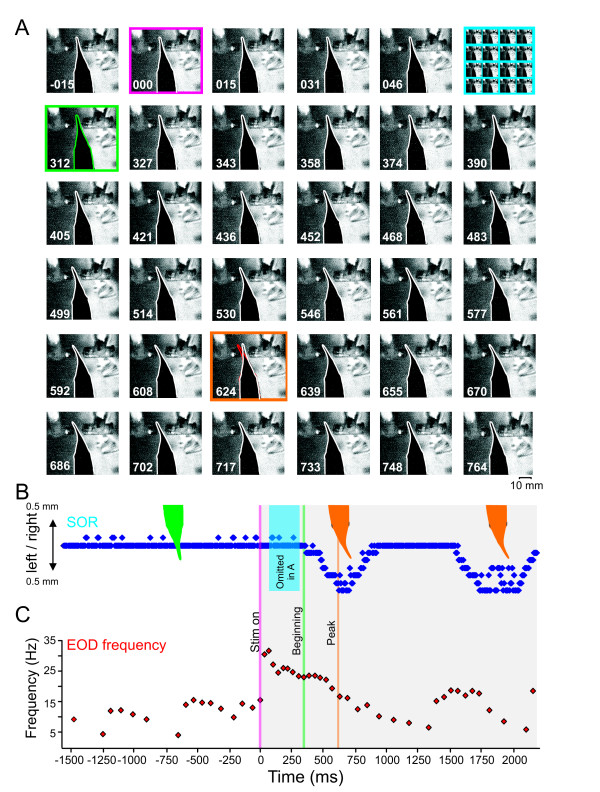
**Movement of the Schnauzenorgan**. **A**. Sequence of the SOR filmed at 128 frames/s ordered from left to right and top to bottom. Every second frame is shown, resulting in a timeline of 15.6 ms from frame to frame. Numbers in each frame give the time in ms with respect to stimulus onset. The dipole-object's resistance was changed during the 2^nd ^frame of the first row. The beginning of the SOR is highlighted by the green square (first frame in the second row). The peak displacement of the SO is indicated by the orange frame. Note that for the sake of clarity, eighteen frames between the stimulus and the initial SOR have been omitted (see top hand corner in A and blue box in B). **B**. Tracking data of the Schnauzenorgan's displacement. **C**. Instantaneous EOD-frequency as measured during the sequence shown in A and B. Note that the SOR peaks about 400 ms after the novelty response. Time of stimulation, first and maximal SOR are indicated in B and C by the three vertical lines.

**Figure 3:**
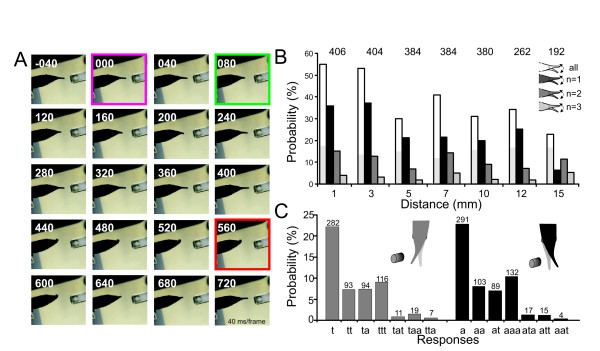
**Probability of the SOR**. **A**. Example of the Schnauzenorgan response evoked at the tip of the Schnauzenorgan. The change in resistivity occurred in the second frame (frame-rate 40 ms/frame, timeline in individual frames is given with respect to stimulus onset). First movement (green) and max SOR (orange) are indicated. **B**. Bar plot of the probability of the occurrence of a Schnauzenorgan response with the object placed at different distances from the skin pooled for all dipole-positions. Only responses that consisted of one (black), two (medium grey) or three full movements (light grey) are shown. White bars show the absolute probability of a response. The light grey area within the white columns represents the probability of a Schnauzenorgan response evoked by a control stimulus (no change in object resistance). This measure served as a reference for the spontaneous level of Schnauzenorgan responses. Data were pooled for *on*- and *off*-stimuli. **C**. Probability of 14 different types of movement observed during an SOR. Light grey columns correspond to movements starting with a movement towards the object, while black columns show those responses where the Schnauzenorgan initially was moved away from the object. The direction of consecutive movements is given by the letters below the bars (a = away, t = towards). Numbers above graphs in B and C give the absolute number of stimuli the data are based upon.

In 14 fishes the responses to sudden changes in the impedance (n = 1206) of a dipole object [14] (Fig. 1) were tested (Fig. 2). Switches were between a shunted and open-circuit object (see standard stimulus in the material and methods section). Motor-responses of the Schnauzenorgan could be evoked in all fish by both, an impedance-switch from a low to a high impedance (Fig. 3B, *on*-stimulus) or when returning from the high impedance state to the low impedance state (*off*-stimulus). While the absolute change in resistance is identical for the *on*- and *off*-stimuli, we currently have no data on the effect of the baseline level, i.e., we do not know if similar contrasts of the stimuli will evoke similar motor-responses regardless of the baseline from which stimuli come from. SORs had a mean angular velocity of 89.4 ± 34.3 deg/sec (data based on 15 responses of two fishes using high-speed video-tracking, range: 41 - 151 deg/sec). Depending on the distance and placement of the dipoles, response probability reached levels of up to 70% (see Fig. 3B, C). Responses to both *on*- and *off-*stimuli were pooled since they occurred at equal probability when the standard stimulus paradigm was applied (χ^2 ^= 0.18, p = 0.67, df = 1). Since either two dipole-objects were placed at both sides of the animal or a single dipole-object was placed in front of the Schnauzenorgan, the observed movement patterns are analysed separately below.

When a single dipole-object was placed frontally to the tip of the Schnauzenorgan (see Fig 3A), the SORs occurred either to the sides, or, especially at the shortest lateral distance tested (1 mm), consisted of a downward retraction of the Schnauzenorgan (Fig 3A).

When the dipole-objects were positioned to the sides of the animal (see Fig. 2A), the initial movement in response to the stimulus could be directed either towards (634 of 2412 stimuli) or away (639 of 2412 stimuli) from the object. Movements in either directions were observed at equal probability (χ^2 ^= 0.66, p = 0.41, df = 1). *On*-stimuli evoked slightly more evasive movements (292 towards, and 432 away), while *off*-stimuli resulted in more movements towards the object (330 towards and 309 away) (χ^2 ^= 3,97, p = 0.046, df = 1).

In general, the Schnauzenorgan moved to or away from the dipole-object, reached a maximal displacement (up to 28 degrees or 5 mm displacement of the tip) and then moved back to the resting position. Here, it either stopped (282 responses towards the object, 291 movements away from the object), continued to move in the opposite direction (n = 94/89), or moved again in the same direction as in the initial response (n = 93/103). Up to seven consecutive movements of the Schnauzenorgan following a single stimulus were observed (see Fig. 3C). However, more than two Schnauzenorgan movements were rarely observed, and their frequency was equal to that to control stimuli (see below). Movements were equally likely directed towards or away from the change in the electric field, i.e., the SORs were undirected.

### Frequency of the SOR in relation to stimulus location

The probability at which a Schnauzenorgan response can be evoked depended on the lateral distance and the position of the dipole-object and varied between fishes. In all cases, the SOR-likelihood was highest when stimuli were delivered close to the skin and declined with increasing lateral distance, reaching the level of the controls (16%, average over all controls) at 15 mm distances (Fig. 3B).

**Figure 4:**
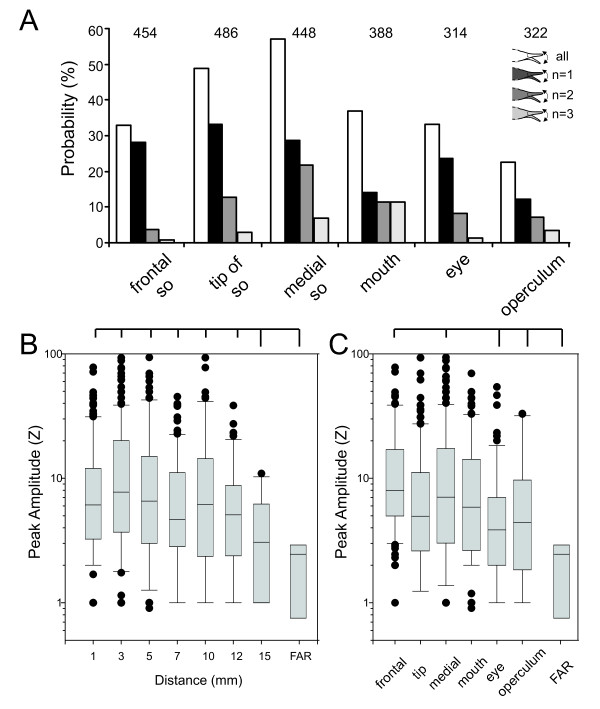
**Probability of SOR**. **A**. Probability of the SOR as a function of the dipole-object's position along the body of the fish. Black filling represents a single SOR, medium grey two, and light grey bars three consecutive responses, respectively. The total probability of a SOR is given by the white bars. Numbers above the graphs show the absolute number of stimuli (100%) at each position. **B**. Box-and-Whisker plot of the amplitude (peak amplitude) of the SOR as a function of distance. SOR amplitude were almost constant at object distances of 1 to 12 mm, while amplitudes decreased at a distance of 15 mm **C**. Box-and-Whisker plot of the peak amplitude of the SOR as a function of dipole position. SORs declined in amplitude form rostral to caudal along the fish's body. In B and C horizontal lines above the plots summarise the results of the Dunn post-hoc test (p < 0.05). Data labelled FAR in B and C indicates the false alarm rate amplitudes.

Analysis of the probability of SORs as a function of the placement of the dipole showed that SORs were evoked most reliably at the middle of the Schnauzenorgan, whereas SOR-probability declined towards the operculum and towards the tip of the Schnauzenorgan (Fig. 4A, C).

### Amplitude of the SOR

A considerable variation in the effectiveness of eliciting SORs was found between fishes, especially with regard to object distance. SORs in response to changes of the impedance of dipoles placed at a distance of 1 to12 mm were of equal amplitude and significantly stronger than those obtained at a distance of 15 mm (Kruskal-Wallis test: h = 20.6, F = 3.51, post-hoc Dunn test p < 0.05; Fig 4B). At 15 mm distance, the amplitude of the movement was indistinguishable from spontaneous movements of the SO (Fig. 4B, false alarm rate - FAR). Spontaneous SORs occurred at a low rate (7 SORs in 384 stimuli) and their amplitude was lower than those caused by dipole-objects placed within a range of 12 mm from the animal's skin (see Fig. 4B, C). Thus, within the range where the animals were responsive, SORs caused by our dipole-objects were of similar amplitude and this range extended up to a distance of about 12 mm from the skin surface.

Combining SORs at all distances for each position shows that the SOR amplitude decreased weakly from rostral towards more caudal dipole positions (Spearman's rho = -0.1, p = 0.004, N = 806; Fig 4C). Overall, stimulation at the Schnauzenorgan and mouth resulted in stronger SORs than stimulation near the eyes, the operculum or those caused by control stimuli (Kruskal-Wallis test, h = 57.9, F = 8.92, post-hoc Dunn test p < 0.05; Fig 4C).

### Latency of the SOR and the novelty response

**Figure 5:**
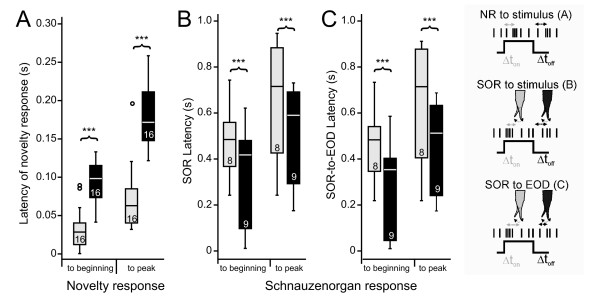
**Temporal aspects of the SOR**. Latency of the novelty response and the Schnauzenorgan response obtained using high-speed video analysis with one fish (128 frames/s). For both responses data are presented with respect to the beginning of a response and with respect to the peak. **A**. Latency of the novelty response for *on*-responses (grey boxes) and *off*-responses (black boxes). **B**. Latency of the SORs with respect to the time of a change in dipole resistance. **C**. Latency of the SORs relative to the time of the first EOD following a change in the dipole-objects resistance. In all cases *on*- and *off*-responses were significantly different from each other (Mann-Whitney U-test: p < 0.01). A legend to the parameters displayed in A-C is shown at the right hand side of the figure.

In order to analyse the latency of the SOR and to compare it with the latency of the simultaneously recorded novelty response, we used high-speed video sequences (128 frames/sec; 16 SORs) obtained from one fish. These data show that the peak of the novelty response occurred at a mean latency of about 40 ms after changing the electrical properties of the dipole-object. The latency of "*off*" responses was significantly longer than that of "*on*" response (paired t-test: p < 0.001)(Figs. 2B, 5A). In contrast to the novelty response, SORs had a much longer latency (about 500 ms, see also Fig. 2 bottom), and "*off*" response latencies were shorter than "*on*" response latencies (paired t-test: p < 0.001)(Fig. 5B, C).

### Sensory modalities evoking SORs

Several experiments were conducted in order to examine which modality of the electrosensory system (active/passive) can evoke SORs. In one series of experiments, the resistance of the dipole-object (3 mm distance, middle of the Schnauzenorgan) was not altered, while the object's capacitance was switched from 1 μF to 1 nF. The effect on the local EOD-amplitude of such purely capacitive switches is equivalent to the effect of a switch from a big resistor to a shunt. However, purely capacitive objects constitute a "filter" for low stimulus frequencies (including DC-potentials) and thus only stimulate tuberous electroreceptors. In contrast, resistive switches can contain low frequencies and therefore potentially stimulate both ampullary and tuberous electroreceptor organs [29].

**Figure 6:**
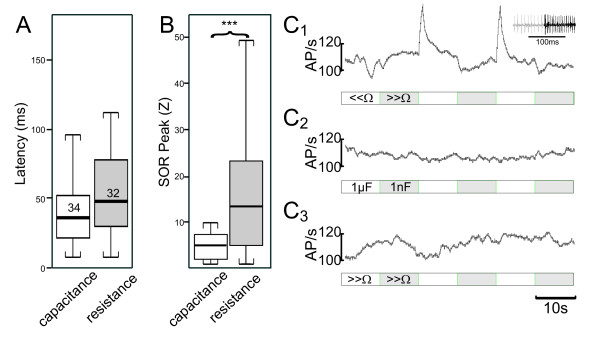
**Controls and ampullary recordings**. **A, B**. Box-and-Whisker plots showing the latency (**A**) and the amplitude (**B**) of the SOR for a single fish presented with changes in capacitance of the dipole-object (white bars, n = 34 stimuli), and mean data of four fishes where stimuli consisted of changes in object resistance (32 stimuli). **C**. Ongoing discharge rate of an ampullary receptor in response to changes in resistance (C1), capacitance (C2) and in response to the control stimuli (no change, C3). Note that the receptor responds with a transient increase in spike rate only to a change in object resistance and that this response is strongest for the return to the shunted dipole-object. The inset in C1 shows a short spike sequence.

Capacitive switches evoked a SOR in 45% of all stimuli (70 switches, 32 SORs). In comparison, purely resistive switches (from a short to an isolator) under otherwise identical conditions lead to SORs in 55 out of 64 cases (86%; 64 switches in 4 fish). SORs caused by capacitive switches were less frequent and also lower in amplitude compared to those caused by purely resistive switches (Mann-Whitney U-test, Z = -2.5, p < 0.011; Fig. 6A). In contrast to these differences, the novelty responses evoked by the two types of stimuli were indistinguishable in amplitude (t-test, t = 1.8, p = 0.08) and latency (t-test, t = 1.70, p = 0.11).

The above data suggests that SORs can be elicited by the active electric sense alone, because purely capacitive switches were sufficient to evoke SORs.

However, the higher likelihood of evoking an SOR with purely resistive switches indicates an additional involvement of the ampullary sensory system in mediating SORs. Recording ampullary receptor activity in response to both purely resistive and purely capacitive changes showed that these receptors (n = 3) responded to purely resistive switches, especially from a shunt to a high resistance (Fig. 6C1). Switching from large to small capacitances, however, did not elicit any changes in receptor activity (Fig. 6C2). Likewise the control, where no change in the resistive load occurred, i.e., switching between two identical resistances, did not result in any reproducible modulations of the ongoing discharge rates (Fig. 6C3).

**Figure 7:**
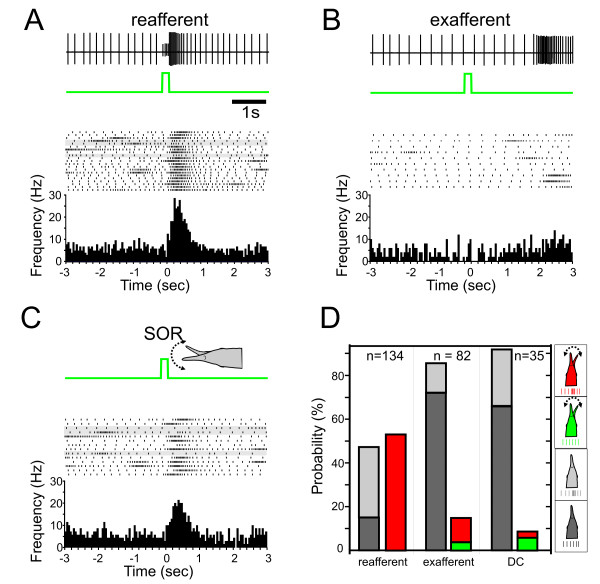
**Ex- and reafferent stimuli and the SOR**. **A, B, C**. Comparison of the EOD pulse behaviour for short switches from 10 Ohm to an open circuit. The response to this stimulus is presented according to whether the switch occurred during the occurrence of EODs (**A**, reafferent) or in between two consecutive EODs (exafferent, **B**). In **C **data are presented for all switches that caused a SOR, irrespective whether the stimulus was ex- or reafferent. The upper trace indicates the recording of the local EOD and the stimulus applied. The raster below the stimulus represents all EODs associated with a given stimulus paradigm, and the histogram at the bottom shows the mean inter-EOD frequency. Stimuli not associated with a novelty response are highlighted in grey in B and C. **D **Bar-plot showing the probability at which stimuli evoked no response (dark grey), a novelty response (light grey), a SOR but no novelty response (green), or both responses (red). Data are based on three fishes. Note that reafferent stimuli were most efficient in evoking SORs and novelty responses, whereas exafferent stimuli and weak DC pulse had an overall low probability for eliciting either form of behavioural responses.

Thus ampullary receptors could potentially contribute to the SOR. To further test if DC-potentials might cause SORs, we conducted experiments, in which the dipole was used to deliver weak DC potentials. If the delivered DC was of comparable amplitude to that measured in response to purely resistive switches, SOR and NR probability were low (see Fig. 7). Hence it is unlikely that the DC-potentials of the resistive switches alone caused motor responses. Further evidence for this comes from supplementary experiments where the EOD amplitude was shunted (decrease of EOD amplitude by 80%). Under these conditions, SORs were still evoked by resistive switches. With such a reduced electric field, the mormyromasts are expected to be unresponsive, so the observed SORs are most likely due to the ampullary system, which is not affected by the shunting.

The above data strongly suggests that the main sensory channel mediating the Schnauzenorgan response is active electroreception. To further substantiate this claim, we compared the responses to short switches (40 or 200 ms duration) that were either presented in between successive EODs or within the time period during which one or several EODs were emitted. Switches between EODs are *exafferent *stimuli and as such only influence the passive electrosensory system, whereas switches including EODs are *reafferent *and thus activate both the active and the passive electrosensory pathways. In accordance with the data shown above, SORs as well as novelty responses were most reliably evoked by *reafferent *stimuli (see Fig 7).

In summary, we have shown that the active electrosensory system is the principal modality leading to Schnauzenorgan responses; however, our data also imply that passive or multimodal electric stimuli might additionally contribute to some degree.

### Comparison to the novelty response

**Figure 8:**
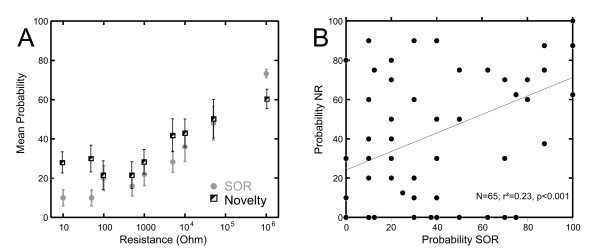
**SOR and Novelty depend on the amplitude change of the EOD**. **A**. Scatter plot of the relationship between the probability of the novelty response (cirlces) and the SOR (squares) with regard to the amplitude of the switch. The baseline resistance prior to any switch was 10 Ohm. Note that both responses scale with the amplitude change of the dipole object, which corresponds to the amount of change in the local EOD amplitude. **B**. Scatter plot of the relation between the mean probability of evoking a SOR and the respective probability of the novelty response. Error bars in A give S.E.M. (N = 7). Data for both figures were obtained with the dipole-object being placed at the middle of the SO at a distance of 5 mm.

In a separate set of experiments (N = 7) we compared the probability of SORs and the amplitude of the novelty response. The stimulus protocol in these experiments deviated from the standard paradigm: switches from a low resistance baseline (10Ω) to pre-defined levels of higher resistance occurred every 6 seconds and lasted only 500 ms. Each step thus resulted in a different amplitude change of the local EOD amplitude. Both the amplitude of the novelty response and the probability of SORs increased with increasing the switch-amplitude (Fig. 8A). This relation only was found for resistive stimuli. Both, novelty response amplitude and the probability of a SOR were low when capacitive stimuli of various strengths were presented, but future experiments are needed to corroborate this preliminary result.

The above data show that both NR and SOR scale in their amplitude and probability with the change of the local EOD-amplitude (*reafferent *stimulus). To investigate if both behaviours must be regarded as independent processes, we next analysed the correlation between SOR-probability and NR-amplitude (Fig 8B). For this analysis, data of several experiments were combined, documenting a weak correlation between both motor responses. This suggests that the two behaviours might occur sequentially and may be regarded as indicative of an early object detection system (NR), followed by an object exploration system expressing itself in an orienting behaviour (SOR) during a period of sustained high sampling of the environment.

**Figure 9:**
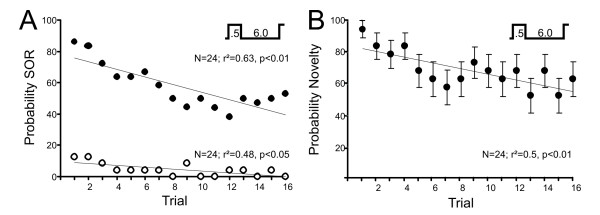
**Habituation of the SOR**. **A**. Probability of evoking the SOR in a series of 16 consecutive trials (inter-trial interval 6 s, see inset). The filled circles show the data for *on *responses, while *off *responses are shown as open circles. Note that stimuli were much shorter than those used for the general description of the SOR, which is reflected in the absence of "off" responses. **B**. Mean amplitude of the novelty response as a function of the number of trials for the data shown in A. In A and B, responses were evoked by a switch that lasted 0.5 s, which was sufficiently short to evoke only a single novelty response and mainly *on-*responses of SORs. Error bars in B are S.E.M.

Previous studies had shown that the novelty response habituates [16] when repetitive stimuli are presented. We therefore tested the effect of repeated stimulation on both the SOR and the novelty response using the same stimulus paradigm as above. Due to the short duration of the stimuli, predominantly *on*-responses were evoked. As expected, novelty responses habituated within a session of 16 stimulus presentations (Fig. 9B). SORs habituated with a comparable time-course, further strengthening the direct relationship between electrosensory stimuli and the SOR (Fig. 9A).

## Discussion

It is well established that some electric fish can respond to changes in their environment with a change in the rate of their EODs [16,19,22,26,30,31]. These novelty responses can be evoked by all sensory modalities. Here we show that novelty responses caused by changes in the impedance of an object located near the Schnauzenorgan can be accompanied by a previously un-described motor-response, the Schnauzenorgan response. Following a novelty response, fish show a quick movement of their Schnauzenorgan towards or away from the object. Both SOR and novelty response probabilities scale with the change in the EOD-amplitude.

Control experiments show that movements of the Schnauzenorgan also occur spontaneously. However, spontaneous movements are weaker (degree of deflection of the Schnauzenorgan) and occur only in about 16% of the control trials and in 2% of the trials used to estimate the false-alarm rate. The comparatively high probability of evoking SORs with the control stimuli probably reflect the presence of the weak DC fields associated with the switches. Responses to real changes of the impedances of the dipole-objects occurred much more reliably. Thus, SORs can clearly be distinguished from spontaneous movements and represent a true sensory-motor response.

### SORs and stimulus location

SORs depended on the rostro-caudal position of the stimulus object as well as on its lateral distance to the skin. The range of about 12 mm within which SORs were evoked above chance level indicates a strong dependency of the SORs on the EOD-amplitude, which declines exponentially with increasing distance. A shunt of the dipole placed 1 mm from the skin increase the local EOD-amplitude by 172%, whereas at 15 mm this increase declined to 7% and to 1% at 25 mm. Based on this data, a threshold of a few percent modulation of the EOD amplitude can be estimated for the SOR. The present analysis suggests that SORs occur as all-or-nothing like events, but a more thorough analysis on the relationship between stimulus location and strength of the SOR based on high-speed videos is needed to confirm this in future experiments.

While our systematic mapping of the response was restricted to the head region of the fish, experiments with the objects placed at the trunk of the fish never evoked SORs. In these experiments, the trunk was only partially covered. We therefore conclude that particularly the Schnauzenorgan is the focal region for evoking SORs, but can not exclude that SORs might also be evoked form other regions in freely behaving animals.

### Which sensory modalities contribute to the SOR?

Novelty responses can be evoked by several sensory modalities and by multimodal stimuli. This contrasts with the SOR, which was only evoked by electrical stimuli. Neither acoustic nor visual stimulation were effective in evoking SORs (but did evoke novelty responses, unpublished observation). We can not rule out, however, that these modalities might evoke SORs under different conditions (e.g., higher intensities). Hydrodynamic or tactile stimuli were not investigated. Given the abundance of electroreceptor organs in the head region, we think it very likely that SORs are only evoked by local electrical stimuli.

### To which extend do the sub-modalities of the electric sense contribute to the SOR?

Both amplitude and waveform modulations were effective in evoking SORs, although amplitude modulations seem to drive the response more efficiently. Mormyromasts have two types of electroreceptor cells, the A- and the B-cells [32]. Theoretically, the response to both resistive and capacitive changes of object properties could be conveyed by B-cell input alone. If input from the A-cell system is also involved in the SOR remains to be clarified.

It is conceivable that weak DC-potential were present when the dipole-objects were shunted or disconnected, which could explain the documented responses of the ampullary system. Switching between two capacitances (1 μF and 1 nF) effectively prevents DC-potentials and hence recordings from ampullary receptor organs were not responsive. However, SORs were still evoked under these conditions, albeit at lower probability. Our experiments on comparing the effectiveness of *ex*- and *reafferent *stimuli in evoking SORs and novelty responses suggest that the predominant stimulus leading to both responses was *reafferent *and thus based on the mormyromast system. *Exafferent *DC-potentials only caused SORs or NR at amplitudes exceeding those due to DC-potentials in our setup. We therefore conclude that the mormyromast system is involved in triggering SORs and that the ampullary system also contributes to it, probably in a multimodal manner. Further experiments addressing the contribution of either modality more directly are needed to resolve this question unequivocally.

### SORs and the Novelty response

The amplitude of the novelty response and the SOR-probability scaled with the change in the local EOD. Thus, SOR probability is correlated with the amplitude of the novelty response. This establishes a perceptual link between both behaviours, where stronger changes in the electric signal will evoke stronger behaviours. Again, our data indicates that EOD-amplitude modulations in the order of a few percent will induce a SOR.

Novelty responses always occurred at shorter latencies than the SOR, usually after the first EOD following a change. The latency of SORs varied between 50 and 280 ms, thus SORs occurred later than the novelty responses. SORs surely are the result of sensory-motor integration on a relatively short timescale, yet take much longer than C-starts or the amazingly short and precise orienting response of Archerfish to their prey [33,34]. This and the fact that SORs were non-directed, indicate that the SOR is not a reflex, which always is unipolar, i.e. an orientation is always to be observed towards the stimuli or an escape from the stimuli.

SOR occurrence also was more localised, with a focus on the Schnauzenorgan region, further emphasising the role of the SO as an electrosensory fovea. SORs were rarely seen when stimulation occurred caudal to the mouth and control stimuli presented at the trunk never elicited SORs, whereas novelties were evoked at all position tested (unpublished observation).

While we have no data concerning the sensory-motor loop involved in the SOR, it is interesting to note that the trigeminal nerve is hypertrophied in Mormyrids [35], including *Gnathonemus*, where a large branch innervates the Schnauzenorgan [36-38]. The presence of a motor response in conjunction with electrosensory input strongly suggests reciprocal innervations between the motor nuclei and sensory areas, which will be worthwhile to investigate further. We speculate that such connections should be present between medullary centres processing direct sensory input from both the electroreceptors and the trigeminal nuclei.

The correlation between the SOR-probability and the NR-amplitude might indicate that both motor behaviours are part of a sequential process that is triggered by the detection of a change in the environment. This initial detection then leads to a novelty response, followed by a SOR provided that the stimulus was strong enough. Thus, one could attribute the NR to an early detection phase during object inspection and the SOR together with an increased EOD-frequency to an exploration phase.

At present it is impossible to decide whether the two behaviours are triggered independently by a stimulus change in the environment or whether they are triggered sequentially, i.e., a novelty response triggers the SOR.

A further parameter that has been investigated regarding the novelty response is habituation. Similar to the novelty response, the SOR does habituate if stimuli are presented repeatedly.

### SORs and natural movements of the Schnauzenorgan

SORs were undirected single movements of the Schnauzenorgan, but up to seven consecutive movements were observed. Theses were reminiscent of the rhythmic scanning movements of the Schnauzenorgan, which these fish perform during foraging [39,40]. Irrespective whether a single or multiple SORs were evoked, SORs were quick, with angular velocities reaching 89.4 ± 34.3°/sec. This value is lower than the natural scanning movement observed in freely moving animals, where values of up to 500°/sec have been reported [28]. However, these data were based on regular video analysis at frame rates of 40 ms and an analysis of freely moving fish using high-speed videos (128 frame/sec) indicates that natural scanning movements are comparable in their speed to evoked SORs.

The chin region of Mormyrids in general is characterised by a high density of electroreceptors, a phenomenon that has been referred to as an electric fovea [27,39]. The rhythmic-scanning movements might help to overcome a severe limitation of such an electrosensory fovea. Reserving highest acuity to a small portion of the sensory mosaic requires orienting mechanisms, which allow the animal to actively explore the environment by moving the foveal region. In foveated vision for example, pursuit eye movements and saccades overcome this limitation. Similarly the scanning movements of the SOR will increase the area that is analysed by the electrosensory fovea.

### Possible functions of the Schnauzenorgan-response

The platypus (*Ornithorhynchus anatinu*s) is an passive electrosensory animal [41], which shows a scanning behaviour with its beak when searching for food. Behavioural experiments on freely swimming platypus have shown that they also turn their head in a reflex-like manner towards new electrical stimuli. In most cases, this head-turning was followed by a complete turning of the body towards the source [42]. Similar to the Schnauzenorgan of *G. petersii*, the electroreceptors of platypus are located on the beak, with the highest receptor organ density at its edges [43].

Yet a different example of electrosensory foveation in the passive electrosensory system can be found in paddlefish (*Polyodon spathula*), which have the highest density of ampullary receptors on their rostrum [44]. These fish swim in an undulatory manner that imparts a lateral oscillating motion to their rostrum. These saccade-like motions of the rostrum are interpreted as an adaptation for prey detection, since they increase the width of the electrical scan field. In contrast to the SOR, however, these movements are rather slow. If a potential prey is encountered, however, a fast and precise turning of the rostrum takes place.

All examples mentioned above perform rhythmic scanning behaviours with an elongated part of their body covered densely with electroreceptor organs (the rostrum, the beak, or the Schnauzenorgan). These areas can be described as electric foveae [27,45,46], which require a scanning mechanism to extend the range of a small aspect of the sensory world beyond the actual aperture of the fovea. Therefore, we consider the rhythmic scanning movements that occur during foraging to constitute an active extension of the area of the electric environment that can be explored by the electric fovea. If the animal encounters a novel object during scanning, the undirected oscillating movements change to active orientation towards the new stimulus. In both the platypus and the paddlefish, this orienting response is highly accurate and based on passive electrolocation. This is different to the SOR of *Gnathonemus*, because these fish also have an active electric sense and therefore additionally employ active electrolocation.

Whether the SOR is guided towards or away from a source might be context-dependent. Alternatively, the SOR might be non-directed because of the relatively small size of the Schnauzenorgan. Due to the small fraction of the electrical field that can be explored with this protrusion, it could be that a quick, source directed turning of the Schnauzenorgan within a DC-field is not possible since it would require an almost instantaneous computation of the electric field in order to determine its source. In contrast to the rostral appendages of the platypus and the paddlefish, the Schnauzenorgan of *Gnathonemus *might be too short and too thin for an accurate estimation of the source's direction. Instead, some kind of "approach algorithm", which involves the comparison of the field intensities at different positions within the field, might be required. Independent from the above argumentation, relative movements are know to enhance the ability to detect DC-potentials by the ampullary system [47].

## Conclusion

We here present the first report of a new motor response to electric stimuli in the weakly electric fish, *Gnathonemus petersii*. This response consists of one or several fast movements of the Schnauzenorgan which are evoked by changes in the nearby electric environment. We show that while both sub-modalities of the electric sense, i.e., active and passive electrolocation, can evoke this motor response, the contribution of the active sense dominates. In contrast to the well-described novelty response, the SOR is of longer latency and is restricted to electric stimuli in the proximity of the Schnauzenorgan. The Schnauzenorgan thus can be considered as an electric fovea serving as an active electrical probe in environmental imaging. Previously, we could show that several adaptations exist that optimise the electric field for active electrolocation [40,48] at this foveal [27,39] appendage.

We propose that since SORs and novelty responses can be evoked in restrained animals, they offer a unique opportunity to extend the study of sensory-motor interaction to electrophysiological studies comparable to the well established eye movements or the vestibular-ocular reflex [49,50]. To this end, a better understanding of the trigeminal system and its interaction with pre-motor areas is required.

## Methods

Twenty-four fish (*Gnathonemus petersii*, Günther 1862), all purchased from Aquarium Glaser (Rodgau, Germany), were used in this study. Fish were housed in two 100 litre tanks of constant temperature (22-24°C) and electrical conductivity (100 to 120 μS/cm).

### General methods

Before animals could be transferred to the experimental set-up, they were anaesthetised by immersion in MS-222 (tricaine methane sulfonate; Sigma, St. Louis, MO, USA; concentration 306.17 μmol l^-1^) and transferred to a holder. Here, the fish's trunk was lightly restrained. The animals head was not covered from the pectoral fin to the SO. The holder was placed in a tank (30 × 19.5 × 18.5 cm^3 ^LxWxH) with fresh water (123 μS cm^-1^), where the fish recovered from anaesthesia within a few minutes.

A video camera (Sony DCR-HC 40E) was placed above the tank and images of the animals were acquired and stored at 22 frames/s with a PC running Viewer^2 ^(BIObserve, Bonn, Germany). A pair of carbon electrodes on the inside wall of the tank between the head and tail of the fish was used to record the electric organ discharges (custom-built amplifier, band pass filtered 10 Hz-10 kHz). EODs were stored using a CED digitiser (Cambridge Electronic Design, Micro 1401 12 bit, 200 kHz, analogue-digital converter) and Spike2 software (Cambridge Electronic Design Ltd).

Stimuli used to obtain a general characterisation of the SOR (standard stimulus paradigm) were delivered by two passive dipole-objects, each consisting of two carbon poles (diameter 5 mm distance between pools: 9 mm) in Perspex tubing in the form of an inverted T. The carbon poles were connected to a switch that allowed altering the resistance or the capacitance between the poles. The dipole objects were placed with the carbon poles oriented perpendicular to the midline of the animal (see Fig. 1). Except for the tip of the Schnauzenorgan, where the orientation of the dipole-object was along the rostro-caudal axis of the animal, two such dipole-objects were placed at equal distances at opposing sides of the animal. Distances between dipole-object and the skin of the fish were 1, 3, 5, 7, 10, 12 and 15 mm, and placement of the objects along the length of the animal is shown in figure 1. If at a given position novelty responses or SOR could no longer be evoked, we did not test the responses at further distances.

The resistance of each dipole-object was changed independently - using a manual switch - from a short circuit to an infinitive resistance (open circuit) and back again to the short circuit condition. These changes in resistivity of the object are referred to as a trial. Trials lasted between three and six seconds and consisted of an *on-stimulus *(initial change in object impedance) and an *off-stimulus *(return to the shunted condition). Note that "on" and "off" refer to an increase and a decrease in the dipole object's impedance, respectively. Between successive trials the dipole-object was kept in the "shunted" condition for a variable length of time (40-80 seconds). Each switch in resistivity of an object generated a trigger pulse that was recorded in Spike2 and Viewer^2^. Responses to stimuli were tested in four fish for all object positions and distances. At each position, eight stimuli and one control stimulus were presented. Controls consisted of no changes in the resistive load, i.e., switching was between two identical resistances. These controls therefore tested for the presence of an animal's response to possible weak mechanical or acoustic stimuli associated with switching, as well as for possible DC-potential effects. Eight consecutive trials together with one control trial are referred to as a session, and a maximum of six sessions were gathered with a single fish per day. Between each session the objects resistances were not altered for 10 minutes.

### Data Analysis

Novelty responses consist of a shortening of the intervals between consecutive EODs. Following methods that enhances the contrast between fluctuations of EOD pulse intervals and accelerations related to stimulation established by Hall and colleagues [19], intervals between successive EODs (Δ) were calculated (). The instantaneous acceleration (*a*) was calculated next as the distance between intervals (*a *= Δ_*i*-1_-Δ_*i*_). The mean acceleration () for the eight trials within one session was calculated. Of these we subtracted the mean acceleration of the 20 EODs prior to a switch and divided this by the standard deviation (*σ*):



The Z-data thus obtained provided a statistical measure of the amplitude of the novelty responses where z-scores below 1.96 occur above chance level (p < 0.05 see Fig. 1B). The maximal deviation of the z-transformed data within 5 EODs after a switch was taken as the peak amplitude of a novelty response. The latency to this peak response was measured both with respect to the time of switching dipole properties and with respect to the first EOD following this change in resistivity. Similarly, we measured the latency to the first significant deviation of the transformed data, i.e., to the beginning of the novelty response.

The videos of the animal's movements were analysed with custom written software (OMA; M. Hofmann, Bonn, Germany). This resulted in a frame-by-frame determination of the displacement of the SO. These position-data were analysed in a similar way as described for the novelty responses, i.e. the position data of the SO were z-transformed. The baseline data used for calculating the means and standard deviations were obtained from the 120 frames (or 4.8 s) prior to every switch. This amount of time proved to be sufficient to make the analysis robust against minor changes of the position of the SO.

From the z-transformed data we determined the amplitude of the Schnauzenorgan-responses (SOR), defined as the peak of the transformed data. The latency of this peak as measured with respect to the stimulus onset as well as to the time of the first EOD following a stimulus. This analysis is restricted to all laterally directed Schnauzenorgan movements, since downward movements of the Schnauzenorgan could not be quantified from our videos. In those cases where high-speed videos (128 frames/s) were obtained, the latency of the response was additionally measured with respect to the start of a response, i.e., the first crossing of the threshold level (see Fig. 2C). The number of individual SORs and their directions in response to a single stimulus were noted, i.e., we determined whether the SO moved towards or away from the dipole object. Likewise, the false-alarm rate (and its amplitude) was calculated for a fictive switch of the dipoles impedance 50 EODs prior to the actual changes of the dipoles impedance.

### Experiments on modalities involved in the SOR and habituation

#### Controls with shunted EODs

In order to diminish the amplitude of the EOD produced by the fish and thereby impair active electrolocation, the tail of the fish was wrapped in aluminium foil. This caused a short circuit of the electrical organ. In these experiments, the two dipole objects were positioned at a lateral distance of 3 mm at the middle of the SO, and novelty responses and SORs were evoked, investigated, and analysed in the same manner as described for the standard paradigm above.

#### Controls using capacitive objects

Experiments were similar to the standard paradigm, except that the switch in object properties was between two capacitances, i.e. a switch from a 1 μF to a 1 nF capacitor. This corresponded to a change from a very large to very small object impedance, but the capacitances constituted a filter for DC currents. As in the first control, these measurements were conducted with the dipole objects positioned at a lateral distance of 3 mm at the middle of the Schnauzenorgan.

#### Controls applying *re*- and *exafferent *stimuli

Experiments were similar to the standard paradigm, but the switch-duration and timing was set by an external waveform generator (Accupulser, WPI). The duration of switches in a range between 40 and 200 ms, depending on the discharge rate of a fish (N = 3), and the inter-switch interval was set to 40 seconds. Probabilities of SORs and NR were scored with regard to whether the switch included one ore more EODs (*reafferent*) or no EODs (*exafferent*). As in the first control, these measurements were conducted with the dipole objects positioned at a lateral distance of 3 mm at the middle of the Schnauzenorgan. In similar experiments the waveform generator was used to apply weak DC-potentials using a stimulus isolation unit (WPI Linear Isolator 395) via the dipole object.

#### Habituation and relation to EOD properties

Experiments addressing habituation of the SOR deviated from the standard paradigm in that switches lasted only 0.5 seconds and occurred at a fixed inter-stimulus interval of 5.5 seconds. This ensured a single novelty response was evoked only. These experiments were conducted with the dipole placed 5 mm away from the medial part of the Schnauzenorgan.

Using the same approach we tested the influence of the amplitude of the switch on the strength and probability of the novelty response and the SOR. In these experiments, 10 consecutive switches from 10 Ohm to defined higher resistances were tested.

#### Recordings from ampullary receptors

These experiments were conducted in a separate tank (27 × 13 × 8 cm^3^, 100 μS cm^-1^) with fish that were curarized and artificially respirated. Fish initially were anaesthetised (MS-222, 306.17 μmol l^-1^) followed by an injection of 30 μl Pancuronium (solution 4 mg Pancuroniumbromid per 2 ml, thinner 1:400, Organo Teknika). Afterwards the fish was rigidly placed in the tank where it was connected to an artificial respiration system. Recording technique and animal handling was similar to that described elsewhere [51].

### Statistics

If not stated otherwise, data are given as means with their standard deviations. All data were analysed for normality and depending on the results, parametric (ANOVA and t-tests) or non-parametric test (Mann-Whitney U-test and Kruskal-Wallis test with Dunn post hoc analysis) were performed. When distributions (tested by chi^2^) or means (tested by t-test) of data for *on *and *off *responses were not different, data were pooled. All statistics were performed using SPSS and Matlab.

## Competing interests

The authors declare that they have no competing interests.

## Authors' contributions

JE and SN carried out most of the laboratory work and data analysis and drafted the manuscript. TR carried out experiments comparing the novelty response and the SOR. JE and VDE designed the study and wrote and finalized the manuscript. All authors read and approved the final manuscript.
